# Astroglial Glutamine Synthetase and the Pathogenesis of Mesial Temporal Lobe Epilepsy

**DOI:** 10.3389/fneur.2021.665334

**Published:** 2021-04-13

**Authors:** Mani Ratnesh S. Sandhu, Benjamin F. Gruenbaum, Shaun E. Gruenbaum, Roni Dhaher, Ketaki Deshpande, Melissa C. Funaro, Tih-Shih W. Lee, Hitten P. Zaveri, Tore Eid

**Affiliations:** ^1^Department of Laboratory Medicine, New Haven, CT, United States; ^2^Department of Anesthesiology and Perioperative Medicine, Mayo Clinic, Jacksonville, FL, United States; ^3^Department of Neurosurgery, New Haven, CT, United States; ^4^Harvey Cushing/John Hay Whitney Medical Library, Yale University, New Haven, CT, United States; ^5^Department of Psychiatry, New Haven, CT, United States; ^6^Department of Neurology, Yale School of Medicine, New Haven, CT, United States

**Keywords:** epilepsy, epileptogenesis, glutamine synthetase, astrocyte, epilepsy network, mesial temporal lobe epilepsy

## Abstract

The enzyme glutamine synthetase (GS), also referred to as glutamate ammonia ligase, is abundant in astrocytes and catalyzes the conversion of ammonia and glutamate to glutamine. Deficiency or dysfunction of astrocytic GS in discrete brain regions have been associated with several types of epilepsy, including medically-intractable mesial temporal lobe epilepsy (MTLE), neocortical epilepsies, and glioblastoma-associated epilepsy. Moreover, experimental inhibition or deletion of GS in the entorhinal-hippocampal territory of laboratory animals causes an MTLE-like syndrome characterized by spontaneous, recurrent hippocampal-onset seizures, loss of hippocampal neurons, and in some cases comorbid depressive-like features. The goal of this review is to summarize and discuss the possible roles of astroglial GS in the pathogenesis of epilepsy.

## Introduction

Astrocytes have historically been thought to serve a primarily structural role by supporting surrounding neurons (1). Over the last 40 years, however, a growing body of evidence has suggested that astrocytes serve important roles in normal brain function, and are critical for axonal growth, energy metabolism, neurotransmitter homeostasis and water/electrolyte balance (2–11). Moreover, abnormal astrocyte function has been postulated to contribute to the pathogenesis of a wide range of neurological and psychiatric disorders (12–17).

Following an acute central nervous system (CNS) injury, astrocytes undergo several morphological and functional changes. These “reactive astrocytes” are present in several pathological conditions While reactive astrocytes were originally thought to reflect scar tissue in response to neuronal injury and loss, recent studies have suggested that reactive astrocytes may in fact play important roles in the causation of many disorders, including epilepsy (18–20).

Glutamine synthetase (GS, also known as glutamate-ammonia-ligase, EC 6.3.1.2), an enzyme that is highly abundant in astrocytes, is of particular interest due to its roles in health and disease (21). Systemic mutations of the GS gene have been associated with brain malformations, seizures, multiorgan failure, and early death (22, 23). Studies have further suggested that acquired GS deficiencies in discrete areas of the brain might play a causative role in various neurological disorders and psychiatric conditions including Alzheimer's disease, hepatic encephalopathy, suicide/depression schizophrenia, and epilepsy (24–32). The goal of this review is to discuss the significance of GS in the pathogenesis of focal epilepsies, particularly mesial temporal lobe epilepsy (MTLE), which is one of the most common types of medication-refractory epilepsies in humans (26, 32–35).

## Glutamine Synthetase and Normal Physiology

GS serves important roles in nitrogen metabolism, acid-base homeostasis, and cell signaling in many species of prokaryotes and eukaryotes (36–38). GS is thought to eliminate or reduce the toxic effects of glutamate and ammonia in the mammalian CNS by metabolizing these compounds (39–41). Moreover, multiple physiological processes, including synthesis of glutamate and GABA, synthesis of proteins, and osmoregulation, rely on a steady supply of glutamine (42). Because GS is the only enzyme capable of synthesizing large amounts of glutamine in the human body, changes in its expression and activity are expected to have significant consequences for normal physiology.

### GS and the Glutamine-Glutamate-GABA Cycle

**Figure 1:**
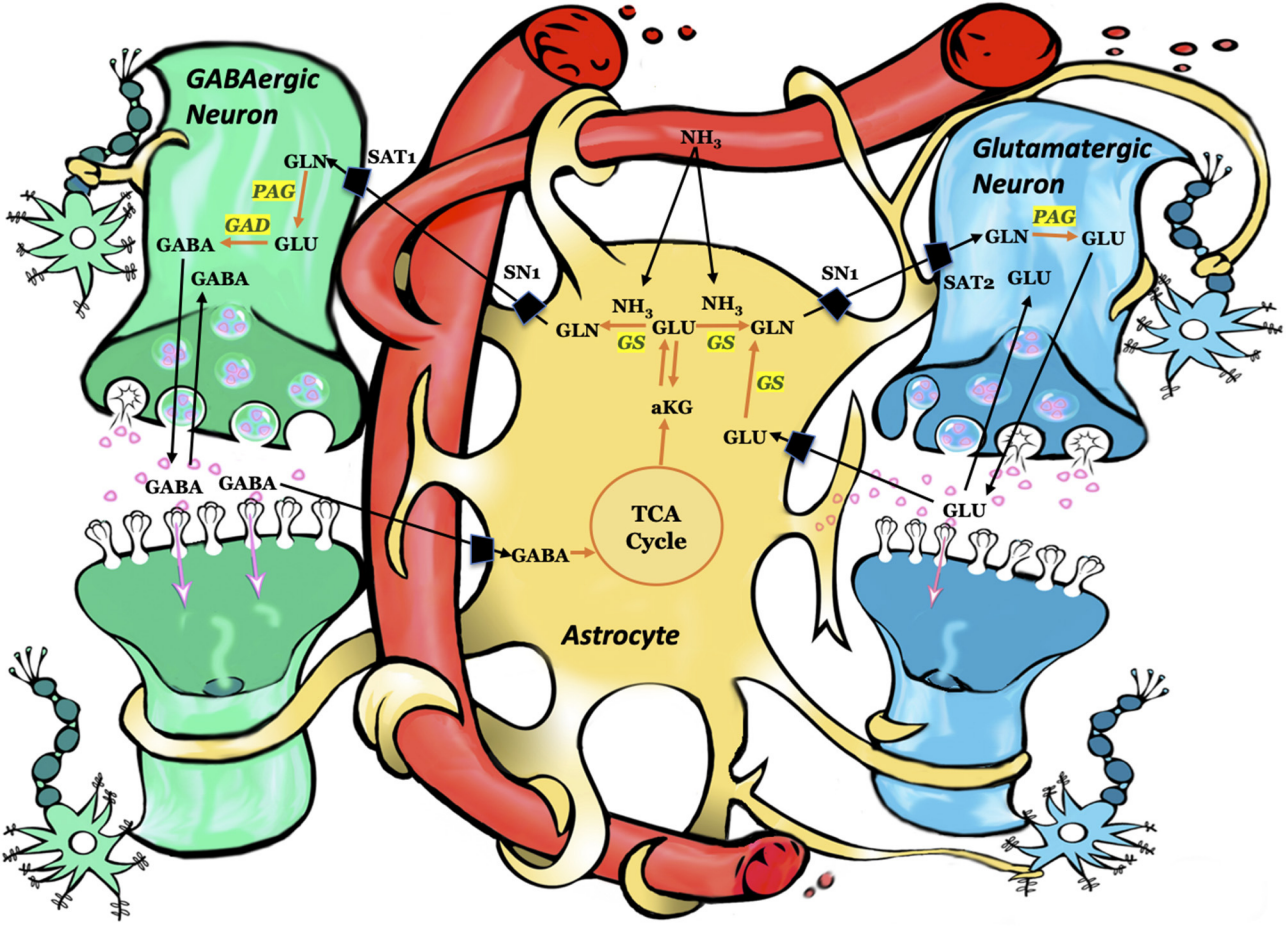
Schematic representation of key pathways involving astrocytes, glutamatergic neurons, and GABAergic neurons. Each arrow signifies several reactions. Using ammonia, glutamine synthetase converts glutamate to glutamine. Subsequently, glutamine is taken up by the adjacent neurons and converted to glutamate or GABA. Astrocytes take up the synaptic glutamate (glutamine–glutamate cycle) or GABA (glutamine–glutamate–GABA cycle) and converts these neurotransmitters to glutamine via glutamine synthetase. GLN, glutamine; GLU, glutamate; GABA, gamma-aminobutyric acid; aKG, alpha ketoglutarate; TCA, tricarboxylic acid cycle; GS, glutamine synthetase; PAG, phosphate-activated glutaminase; GAD, glutamic acid decarboxylase; SN1, system N transporter 1; SAT1, system A transporter and SAT2, system A transporter 2. Figure adapted with permission from (43).

After its release from the presynaptic neuron, neurotransmitter glutamate interacts with receptors in the postsynaptic membrane, followed by its removal from the synapse into astrocytes. After uptake into astrocytes, glutamate may be converted to glutamine via glutamine synthetase (Figure 1). Glutamine can subsequently be transferred from astrocytes to glutamatergic neurons (44, 45). Once in the glutamatergic neuron, the mitochondrial enzyme phosphate-activated glutaminase (PAG) converts glutamine to glutamate (46, 47), which is concentrated in synaptic vesicles and subsequently released into the extracellular space in response to an action potential. The glutamate is either taken up by astrocytes and converted back to glutamine via GS or taken up by inhibitory neurons and metabolized to GABA, which is used during inhibitory neurotransmission (Figure 1). Astrocytic glutamine can be taken up by GABAergic neuron as precursor for the inhibitory neurotransmitter γ-aminobutyric acid (GABA) via glutamate (48, 49). While most of the released GABA is taken up by the pre-synaptic neuron, some enters astrocytes where it is converted back to glutamine (50). Thus, astroglial GS is crucial for both excitatory (glutamatergic) and inhibitory (GABAergic) neurotransmission (51).

### GS and Ammonia Detoxification

The human body contains large amounts of ammonia, produced mainly by the action of bacterial enzymes on colonic content and from the hydrolysis of glutamine in the small and large intestinal cells. While the gut-derived ammonia is mostly metabolized by the liver, via the urea cycle and the GS reaction, a small amount of ammonia (10–30 μmol/L) remains in the plasma under normal conditions. Much higher plasma ammonia concentrations are detected in pathological conditions such as liver disease and urea cycle disorders. Because ammonia is neurotoxic and easily crosses the blood-brain-barrier, an efficient mechanism for clearing brain ammonia is essential (52). Astroglial GS is critical for such clearance, because the CNS lacks a functional urea cycle (52). GS utilizes ammonia, that is taken up by astrocytes, to convert glutamate to glutamine (Figure 1). The important role of astrocytes in this process is underscored by the presence of astrocytic end-feet surrounding the brain endothelial cells, which serve as a metabolic buffer between the blood and the brain, thereby reducing the toxic load of ammonia on neurons (53–55).

## Glutamine Synthetase and Mesial Temporal Lobe Epilepsy

Glutamate is the predominant excitatory neurotransmitter in the adult brain, and perturbed extracellular brain glutamate levels have been implicated in the pathogenesis of epilepsy, particularly MTLE. Notably, extracellular glutamate is chronically elevated in the epileptogenic hippocampus (the seizure onset area) in human patients with MTLE, as ascertained by simultaneous depth electrode EEG and *in vivo* brain microdialysis (56–59). In addition, during seizure activity, a six-fold increase in the extracellular hippocampal glutamate above the basal (chronic) level was observed, followed by a slow decline to basal levels over a period of several minutes (60). Furthermore, many animal studies have suggested a causational relationship between increased brain glutamate signaling and epilepsy (39, 61, 62). Therefore, it is possible that an excessive amount of extracellular glutamate in the seizure onset area of the brain acts as a central metabolic cause of the neuronal loss and spontaneous seizures associated with MTLE (39, 61, 62).

Because GS is thought to be critical for glutamate metabolism following uptake into astrocytes, deficiency in this enzyme has been postulated as a possible basis for the increased glutamate observed in the extracellular fluid of the epileptogenic areas of the brain (26, 62). Moreover, isotopic tracer (C^13^) studies suggest that a slowing of the glutamate-glutamine cycle metabolism in the epileptogenic hippocampus is responsible for the accumulation and reduced clearance of glutamate in MTLE (63). Given the essentiality of GS in the glutamate-glutamine cycle, we and others sought to quantify activity of astroglial GS in the epileptogenic hippocampus in human patients with MTLE (26, 32). Intriguingly, the protein content and functional activity of GS was reduced by ~40% in subfields of the epileptogenic hippocampal formation in patients with MTLE and concomitant mesial temporal sclerosis (26, 32). Similarly, GS deficiency in the amygdala occurs in some patients with neocortical epilepsies (64) and in the tumor tissue of patients with malignant gliomas and secondary epilepsy (65).

Furthermore, several other studies support the idea that GS deficiency is a causative or contributing factor in some types of epilepsies besides MTLE. In the first three known cases of congenital, homozygous mutations in the GS gene, the patients had severe brain malformations and epileptic seizures (22, 66). Two of the patients died shortly after birth (22). Similarly, the high morbidity and mortality rates that accompany genetic GS deficiencies in humans are also observed in transgenic mouse models. For example, animals with prenatal excisions of the GS gene in all cell types do not survive past early embryonic development (67), and mice with selective gene deletions in GFAP-positive astrocytes survive until postnatal day 3 (68). However, mice with selective deletions in the neocortex and hippocampus are born without any apparent malformations but develop neurodegeneration and spontaneous seizures that seem to increase in frequency with age (34).

A common approach to assess the effect of GS deficiencies in specific brain regions involves the use of methionine sulfoximine (MSO). MSO impedes the ability of GS to catalyze the conversion of glutamate and ammonia to glutamine by irreversibly binding to the catalytic sites of GS (69). In one study, a continuous infusion of MSO into the right hippocampal formation of normal adult Sprague-Dawley rats was compared to a continuous infusion of normal saline into the same region of a separate group of rats. The animals were monitored by continuous video-intracranial electroencephalogram (EEG) recordings for several weeks and the brains were analyzed for GS activity. MSO resulted in a reduction of GS activity in the infused hippocampal formation of ~80% (28), and the animals displayed repetitive seizures that began several hours after the onset of infusion. The initial repetitive seizures, which lasted between 24 and 48 h, were followed by a quiet period of variable length before spontaneous, recurrent seizures commenced (70). The MSO-treated animals sometimes exhibited glial proliferation and patterned neuron loss in the infused hippocampal formation, similar to that of human MTLE (28, 33).

Another study analyzed whether the neuroanatomical site of GS inhibition is an important determinant for the epileptogenic process and for the epileptic phenotype. MSO was infused unilaterally into different limbic regions of adult rats, including the angular bundle, the deep entorhinal cortex, area CA1, the molecular layer of the subiculum, the hilus of the dentate gyrus, the lateral ventricle, and the central nucleus of the amygdala (71, 72). Recurrent seizures were observed in all animals infused with MSO into the brain tissue, and the seizures increased in severity (Racine grade) over a period of several weeks with variations in the seizure frequency and severity patterns between brain regions. Moreover, animals infused with MSO into the central nucleus of the amygdala displayed recurrent seizures with depressive-like behaviors, as observed by a reduction of sucrose consumption in the sucrose preference test (72). Collectively, these studies suggest that the neuroanatomical site of GS inhibition affects the epileptogenic process as well as the overall phenotype of the disease.

## Glutamine Synthetase and Epilepsy Networks

The prevalent idea that the neuroanatomical and electrophysiological substrates of focal epilepsies occur only in a circumscribed brain region, the seizure focus, has been questioned more recently (73–77). Many clinical and EEG studies have suggested that large-scale, aberrant “brain networks” play key roles in seizure initiation and propagation, and that aberrant network activity may be present even during the time between seizures (76–85).

Albright et al. analyzed changes in neuronal networks during epileptogenesis in the intrahippocampal MSO-infusion model of MLTE (86). Intracranial EEG recordings and c-Fos immunohistochemistry were used to record seizure-associated neuronal activation at different stages during epileptogenesis. It was found that low-grade seizures during the earliest stages of epileptogenesis activated neurons in the entorhinal-hippocampal territory, the basolateral amygdala, the piriform cortex, the midline thalamus, and the anterior olfactory area. However, during later stages of epileptogenesis, when the seizures were more severe, neuronal activation was evident in extensive areas of the brain, such as the neocortex, the bed nucleus of the stria terminalis, the mediodorsal thalamus, and the central nucleus of the amygdala. These areas were activated in addition to the areas activated during early epileptogenesis.

In another study, whole brain diffusion tensor imaging (DTI) was used to assess any structural changes during early and late epileptogenesis in the hippocampal MSO-infusion model of MTLE (33). There were significant changes in fractional anisotropy (FA) in multiple brain regions in MSO-infused vs. phosphate buffered saline (PBS)-infused control animals. The changes in FA were markedly different in early epileptogenesis compared to late epileptogenesis, suggesting that inhibition of GS in one hippocampal formation results in structural changes that affect multiple brain regions and change over a period of several weeks (33, 71, 86).

## Conclusion

An increasing number of studies in humans and animal models have linked astroglial GS dysfunction to the pathogenesis of focal epilepsies, particularly MTLE. Even very small and circumscribed deficiencies in GS affecting a subfield of the hippocampal formation or a nucleus of the amygdala can lead to epileptic seizures and comorbid, depressive-like features in laboratory animals. Moreover, such small deficiencies result in widespread and progressive changes in neuronal network activation and in the structure of the brain. While the mechanism by which GS dysfunction causes epilepsy remains unclear, several scenarios are possible such as reduced clearance of extracellular brain glutamate and ammonia, glutamine deficiency, and perturbed glutamatergic and GABAergic neurotransmission. Further studies are needed to determine the causes of astroglial GS deficiency so that more effective treatments can be developed to prevent such deficiencies and combat the development and progression of GS-associated epilepsies.

## Author Contributions

All authors listed have made a substantial, direct and intellectual contribution to the work, and approved it for publication.

## Conflict of Interest

The authors declare that the research was conducted in the absence of any commercial or financial relationships that could be construed as a potential conflict of interest.
